# Cryptosporidiosis – an occupational risk and a disregarded disease in Estonia

**DOI:** 10.1186/1751-0147-56-36

**Published:** 2014-06-05

**Authors:** Brian Lassen, Marie Ståhl, Heidi L Enemark

**Affiliations:** 1 Institute of Veterinary Medicine and Animal Sciences, Estonian University of Life Sciences, Kreutzwaldi 62, 51014 Tartu, Estonia; 2 Technical University of Denmark, DK-1870 Frederiksberg C, Denmark

**Keywords:** *Cryptosporidium parvum*, Estonia, Genotype, Human, Zoonoses, Cattle

## Abstract

**Background:**

Cases of cryptosporidiosis have not been officially reported in Estonia after the year 2000, and the disease appears to be either under-diagnosed or under-reported.

**Findings:**

Based on a human case of cryptosporidiosis contracted during faecal sampling in dairy farms, cattle considered to be sources of infection were analysed for *Cryptosporidium* spp. by a modified Ziehl Neelsen technique and molecular typing. *C. parvum* subtype IIaA16G1R1 was detected from the human case and from calves from one of nine farms enrolled in the study providing strong circumstantial evidence of zoonotic transmission from calves to humans.

**Conclusion:**

Cryptosporidiosis presents an occupational risk to people with cattle contact, and may also be a risk to the human population in general. Thus increased public and medical awareness is warranted.

## Findings

Cryptosporidiosis is a diarrhoeal disease caused by the multiplication of protozoan parasites in the small intestine. In humans, cryptosporidiosis is most commonly caused by either *C. hominis*, which is predominantly host specific, or the zoonotic *C. parvum* which is highly prevalent in young calves and much less host specific [1]. In 2007, environmentally robust *C.* spp. oocysts were found in 84% of Estonian dairy cattle herds [2]. Despite the fact that the parasite is shed by 24% of calves <3 months of age it is rarely diagnosed or treated [2,3].

The European Centre for Disease Prevention and Control (ECDC) has stated that food- and waterborne pathogens causing diarrhoea, such as *C.* spp., are of increasing importance [4]. Further it is stressed that improvement of this situation is impaired by under-diagnosing and under-reporting. In Estonia, no new cases of cryptosporidiosis have been reported by the National Health Board after year 2000 [5], although cases have been present as described here. This lack of reported cases of cryptosporidiosis is surprising considering the numerous Estonian cases of giardiasis [4], caused by a protozoan parasite with transmission routes similar to *Cryptosporidium*.

To increase awareness of this issue we present a case of cryptosporidiosis in a 32 year old man with no history of chronic diseases or previous *Cryptosporidium* infection. The man visited nine randomly selected dairy farms in the Estonian counties Harjumaa, Läänemaa, and Saaremaa during a period of three days in January 2007. As part of a university research team, he singlehandedly collected faecal samples from 49 young calves in the farms, and was the only member of the research team who became clinically infected with *Cryptosporidium*. During the period from ten days prior to the last farm visit until the symptom debut, he reported no animal contact besides contact with the study farm, nor did he work with any other faecal samples. The symptoms included stomach cramps, nausea, loss of appetite, fatigue, muscle aches, fever, and malodorous, watery diarrhoea. The clinical symptoms first appeared four days after the last farm visit, and continued with varying intensity for a total of 19 days. Faecal samples taken on days 6 and 14 after the last farm visit, during the most severe diarrhoeal period, were studied using a semi-quantitative, modified Ziehl-Neelsen staining technique [6]. On day 6 and 14, respectively, 1–5 and >25 *Cryptosporidium* oocysts were observed per field of vision at a magnification of 400. Using a commercial kit (E.Z.N.A.® Stool DNA Kit, Omega Bio-Tek Inc.), DNA was extracted from both of these human samples (n = 2), and from *Cryptosporidium* positive bovine samples from calves aged <3 months (n = 3), 3–12 months (n = 3), and >12 months n = 3) representing nine different farms that could have acted as a potential source of the human infection. Samples for genotyping were selected on the basis of *Cryptosporidium* oocysts being present in numbers above 100,000 per gram faeces. The DNA was subsequently submitted to the National Veterinary Institute in Denmark for molecular analysis.

Identification of *Cryptosporidium* to the species level was done by polymerase chain reaction (PCR) amplification and sequencing of the small subunit ribosomal RNA gene (18S rDNA locus) and the 70-kDa heat shock protein gene (*HSP70*) as previously described [7,8], and subtyping was performed using a nested PCR to amplify a ~550 bp fragment of the hyper variable glycoprotein (gp) 60 gene [9]. Subtypes were named according to nomenclature described by Sulaiman et al. [10]. *Cryptosporidium* isolated from the human case as well as from one calf from Raplamaa were identified as *C. parvum,* and 99-100% identical GenBank sequences at the 18S rDNA locus and *HSP70* gene respectively (e.g. AF093493.1; AB542125). Subtyping revealed subtype IIaA16G1R1 [GenBank: KJ769462, KJ769463].

This subtype has mainly been described in calves from Eastern Europe [11-15] but cases in other production animals such as lambs [16] and pigs [17] have also been reported from this part of Europe, and human cases have been described in Slovenia [14] suggesting that this strain may serve as reservoir for human infections.

A physician was consulted and presented with the evidence of clinical cryptosporidiosis 19 days after the initial calf-contact. It then became clear that normal procedure in such gastroenteric cases is symptomatic treatment without any attempts of making a specific diagnosis. In September 2008, the case was reported to health professionals as a work related infection in an occupational health check.

One third of examined Estonian cattle has been shown to excrete *Cryptosporidium* oocysts, and both *C. parvum* and *C. andersoni* have been identified in the population [18]. In 2009, an Estonian study showed that the highest proportion of calves shedding oocysts was seen in animals above 12 months of age, while the highest intensity of infection was observed in calves younger than 3 months [2]. *Cryptosporidium* isolates from this study were not genotyped, however, other studies have shown a strong correlation between age and species/genotype e.g. [19,20], which is highly relevant as regards risk of zoonotic infection since young calves primarily shed the zoonotic *C. parvum* whereas older cattle excrete more host specific species. It is thus expected that transmission of *Cryptosporidium* may occur from young calves to humans, particularly those occupied in the livestock industry, and the present case is in agreement with similar observations in other countries [1,21,22]. Although not officially reported, several veterinary students attending farm visits arranged by the Estonian University of Life Sciences have experienced clinical symptoms consistent with cryptosporidiosis after visiting cattle farms in Estonia.

Cryptosporidiosis is particularly dangerous for young and immunosuppressed individuals. Zoonotic transmission as well as contact with family members suffering from cryptosporidiosis present risks to human immunodeficiency virus (HIV) patients for contracting the disease [23,24]. In Estonia HIV/AIDS is considered epidemic, peaking in 2001 [25]. In 2008, the cases per 100,000 inhabitants reached 40.6 and 4.6 for HIV and AIDS cases respectively [25]. In conclusion, the present situation in Estonia calls for increased awareness towards cryptosporidiosis as a general and an occupational health problem.

## Competing interests

The authors declare no conflict of interests.

## Authors’ contributions

BL presented the idea of the study, collected samples, analysed samples, and drafted the manuscript. MS and HLE carried out molecular analysis and participated in the writing of the manuscript. All authors accepted the final version of the manuscript. All authors read and approve the final manuscript.
